# Immunization with the lipoprotein FtsB stimulates protective immunity against *Streptococcus pyogenes* infection in mice

**DOI:** 10.3389/fmicb.2022.969490

**Published:** 2022-08-09

**Authors:** Li-Yuan He, You-Bo Yu, Ying Liu, Yao-Jin Le, Sha Li, Xiao-Yan Yang

**Affiliations:** Zhuhai Key Laboratory of Basic and Applied Research in Chinese Medicine, Department of Bioengineering, Zhuhai Campus of Zunyi Medical University, Zhuhai, China

**Keywords:** *Streptococcus pyogenes*, lipoprotein FtsB, vaccine, immunogenicity, protective immunity

## Abstract

*Streptococcus pyogenes* is one of the main pathogenic bacteria that causes disease in humans. It is reported that over 18 million cases of *S. pyogenes* disease occurred in the world, and more than 500,000 deaths occur annually worldwide. An effective vaccine is widely regarded as the most reliable way to control and prevent streptococcal infections. However, there is currently no approved vaccine for *S. pyogenes*. In this study, we evaluated the potential of lipoprotein FtsB as a new vaccine candidate to prevent *S. pyogenes* infection. Mice vaccinated with purified FtsB protein elicited high titers of IgG, IgG1 and IgG2a antibodies in mouse serum. Vaccinated with FtsB can reduce bacterial systemic dissemination in the blood, heart, and spleen and reduce organ damage in the mouse bacteremia model. In addition, active immunization with FtsB protected against streptococcal abscess formation. Furthermore, immunization with FtsB was efficient in inducing a mixed cellular immune response and promoting the maturation of dendritic cells in mice. The lipoprotein HtsA was served as a positive control because it has been reported to protect mice from *S. pyogenes* infection in both active and passive immunization. These findings demonstrated that lipoprotein FtsB may serve as a candidate vaccine for the prevention of *S. pyogenes* infection.

## Introduction

*Streptococcus pyogenes* is a gram-positive bacterium, which can produce a variety of toxins (streptolysin, scarlet fever toxin), lipoteichoic acid, streptokinase and other pathogenic factors, thereby causing a series of skin and mucous membrane infections and other diseases, such as pharyngitis, scarlet fever, pustular skin disease, invasion sexual deep tissue infection, and toxic heat shock syndrome, which are a major threat to human health (Dietrich and Steele, 2018). It is reported that over 18 million cases of *S. pyogenes* disease occurred in the world, resulting in more than 500,000 deaths annually, and most of these diseases occur mainly in low- and middle-income countries or regions (Carapetis et al., 2005; Commons et al., 2014; Vekemans et al., 2019). Due to the emergence of *S. pyogenes* resistance to erythromycin, clindamycin, and lincosamide and reduced sensitivity to beta-lactam antibiotics, it is becoming increasingly difficult to cure *S. pyogenes* disease (Seppala et al., 1992; Hasenbein et al., 2004; Hanage and Shelburne, 2020; Musser et al., 2020; Vannice et al., 2020; Johnson and LaRock, 2021). Thus, vaccination is regarded as the most reliable method to prevent *S. pyogenes* infections and reduce the global *S. pyogenes* disease burden, yet there is currently no approved *S. pyogenes* vaccine (Dale et al., 2016; Vekemans et al., 2019).

For several decades, current *S. pyogenes* vaccine strategies have mainly focused on M protein-based vaccines and non-M protein-based vaccines, such as GAS carbohydrate, streptococcal protective antigen, streptococcal pyrogenic exotoxin, and lipoproteins (Dale et al., 2016; Vekemans et al., 2019). Lipoproteins are a class of cell membrane-anchored proteins that are widely present in gram-negative and gram-positive bacteria and have a variety of biological functions, acting not only as bacterial virulence factors but also recognizing and activating the host’s immune system; thus, lipoproteins have become one of the most popular drug targets and vaccine candidates (Kovacs-Simon et al., 2011; Yang et al., 2020).

Iron is an essential element for bacterial growth and infection, and several iron-related lipoproteins have been investigated as vaccines, such as the iron-regulated surface determinants IsdA and IsdB (Kuklin et al., 2006; Kim et al., 2010), the streptococcal haem-binding lipoproteins Shp (Zhang et al., 2017) and HtsA (Song et al., 2020). In *S. pyogenes*, there are three iron uptake systems involved in iron uptake, including HtsABC, FtsABCD, and MtsABC (Janulczyk et al., 2003; Lei et al., 2003; Hanks et al., 2005). The lipoprotein FtsB is a substrate-binding protein of the ferrichrome transporter FtsABCD, which is highly conserved in *S. pyogenes* (Hanks et al., 2005). Moreover, the homologous proteins of FtsB protein also found in other bacterial species, including *Streptococcus dysgalactiae* subsp. equisimilis, *Streptococcus parauberis*, *Streptococcus sanguinis*, *Streptococcus suis* (Supplementary Table 1). The lipoprotein HtsA of the haem transporter HtsABC was reported as a candidate antigen for vaccine development (Song et al., 2020). In addition, the immunogenicity of the lipoprotein MtsA (SPy0453) from MtsABC system is weak (Mortensen et al., 2016). Thus, in this study, FtsB was purified and formulated with an aluminum adjuvant, and its immunogenicity and protective efficacy were determined in mouse bacteremia infection and skin infection models following challenge with the *S. pyogenes* MGAS5005 strain.

## Materials and methods

### Ethics statement

Six-week-old female BALB/c mice were purchased from Beijing Huafukang Biotechnology Company. All animal experiments were approved by the Committee on the Use of Live Animals in Teaching and Research of Zunyi Medical University and performed in accordance with the institutional and governmental guidelines and regulations.

### Bacterial strains, media, and growth conditions

*S. pyogenes* MGAS5005 was grown in 0.5% THYE medium (Todd-Hewitt broth (Oxoid, United Kingdom) containing 0.5% yeast extract (Oxoid, United Kingdom) at 37°C and 5% CO_2_. *Escherichia coli* strains BL21 (DE3) (for the pGEX-4T-*ftsB* recombinant expression plasmid) and Top10 (for the pBAD/HisA-*htsA* recombinant expression plasmid) were grown in Luria-Bertani (LB) broth with 100 μg/mL ampicillin at 37°C with shaking at 200 rpm.

### Protein expression and purification

For the expression and purification of the recombinant protein FtsB, the *E. coli* BL21 strain (DE) containing the recombinant expression plasmid pGEX-4T-*ftsB* was cultured in LB medium and induced with 0.5 mM isopropyl-b-D1-thiogalactopyranoside (IPTG) for 6 h at 37°C. The GST-FtsB fusion protein was purified by GST affinity chromatography, and the GST tag was removed with thrombin exonuclease to obtain the antigen protein FtsB.

For expression of the recombinant protein HtsA, the *E. coli* Top10 strain containing the recombinant expression plasmid pBAD/HisA-*htsA* was cultured in LB medium and induced with 0.04% L-(+)-arabinose for 6 h at 37°C. HtsA was purified by Ni-NTA affinity chromatography. The purity of the FtsB and HtsA proteins was analyzed by SDS-PAGE.

### Animal immunization and antibody detection

BALB/c mice (*n* = 10 per group) were immunized subcutaneously in the back with 20 μg of FtsB in 90 μL PBS that were formulated with 10 μL aluminum hydroxide gel adjuvant (InvivoGen, France) on days 0, 14, and 28. Mice immunized with 20 μg of HtsA protein plus aluminum adjuvant served as a positive control, and mice immunized with 100 μL PBS plus aluminum adjuvant served as a negative control. Blood samples were collected from the tail vein on days 0, 14, 28, and 35 for the enzyme-linked immunosorbent assay (ELISA) of antigen-specific antibodies. Wells of 96-well ELISA plates were coated with 5 μg/mL FtsB or HtsA protein in 50 mM carbonate coating buffer (pH 9.5) overnight at 4°C and then blocked with 200 μL of 5% milk at 37°C for 2 h. Five-fold serially diluted mouse serum samples were added to each well and incubated at 37°C for 1 h. The horseradish peroxidase (HRP)-conjugated goat anti-mouse IgG (Thermo, United States, 1:5,000), IgG1 (Thermo, United States, 1:1,000) and IgG2a (Thermo, United States, 1:1,000) were used as secondary antibodies and incubated at 37°C for 1 h. TMB solution was added to induce the color reaction, and the reaction was stopped with 100 μL of ELISA stop solution. Finally, the absorbance was read using a microplate reader at 450 nm.

### Murine bacteremia model

Seven days after the third immunization (day 35), immunized mice in each group were injected intravenously in the tail vein with 5 × 10^6^*S. pyogenes* MGAS5005 strain. For bacterial burdens and histopathology analysis, blood was collected at 6 h post- infection, and the heart, liver, spleen, lung and kidney were collected at 48 h after the challenge, then fixed with 4% paraformaldehyde at room temperature for 24 h and used for Gram staining and HE staining.

### Murine skin infection model

The immunized mice were anesthetized with pentobarbital sodium followed by subcutaneous injection of 20 μL sterile PBS-diluted bacterial solution containing 2 × 10^7^ *S. pyogenes* on day 35. The body weight and abscess size of the infected mice were recorded every day for 9 days. The skin tissues of infected mice were collected 48 h after the challenge, then fixed and stained with HE, Masson’s trichrome and immunohistochemistry (IHC).

### Cytokine assay

To test the cytokines, mice were sacrificed on day 35 and splenocytes were collected from each group and stimulated with 10 μg/mL FtsB or HtsA. Interleukin 2 (IL-2)-, IL- 4-, IL- 6-, IL- 10-, IL-17A- and gamma interferon (IFN-γ)-producing splenocytes from FtsB-vaccinated or positive or negative control mice were analyzed using corresponding mouse cytokine-specific ELISA kits (Biolegend, United States) according to the manufacturer’s instructions. The results were measured at OD_450_, and concentrations in pg/mL were calculated according to the standard curve.

### Flow cytometry assay

To detect the dendritic cell (DC) cell subsets, splenocytes and homogenates were collected from mice immunized with FtsB, HtsA or PBS on day 35 and used for flow cytometry analysis. Briefly, splenocytes from mice were collected and adjusted to a concentration of 1 × 10^6^ cells/mL. Then, 100 μL cell suspension (1 × 10^5^ cells/mL) from each mouse was harvested and incubated with the fluorescently labeled antibodies FITC-anti-CD11c (Biolegend, United States, 1:200), PE/Cy5 anti-mouse MHC II (Biolegend, United States, 1:200), and PE anti-mouse CD80 (Biolegend, United States, 1:200) at 4°C for 30 min and then analyzed using flow cytometry. The proportion of CD11c + DC cells expressing MHC II and CD80 molecules was calculated.

### Statistical methods

All data in the paper are expressed as the means ± SEM. Differences between multiple groups were analyzed using one-way ANOVA followed by least significant difference (LSD) in GraphPad Prism 5 software (San Diego, CA, United States). Significance is indicated by *p*-values of < 0.05 (*), < 0.01 (^**^) and < 0.001 (^***^).

## Results

### The expression and purification of FtsB and HtsA proteins

**FIGURE 1:**
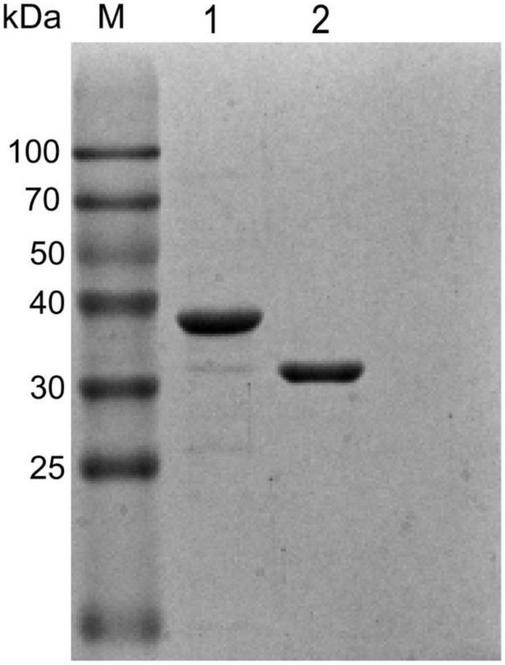
Purified recombinant FtsB and HtsA proteins. The purity of the FtsB and HtsA proteins was analyzed by SDS–PAGE. M represents the protein marker, line 1 represents the HtsA protein, and line 2 represents the FtsB protein.

Recombinant FtsB and HtsA proteins were expressed in *E. coli* BL21 and Top10 and purified using GST affinity chromatography and Ni-NTA affinity chromatography, respectively. As shown in Figure 1, the purity of the FtsB and HtsA proteins was more than 95%, which can be used in further experiments.

### Immunogenicity of FtsB protein in mice

**FIGURE 2:**
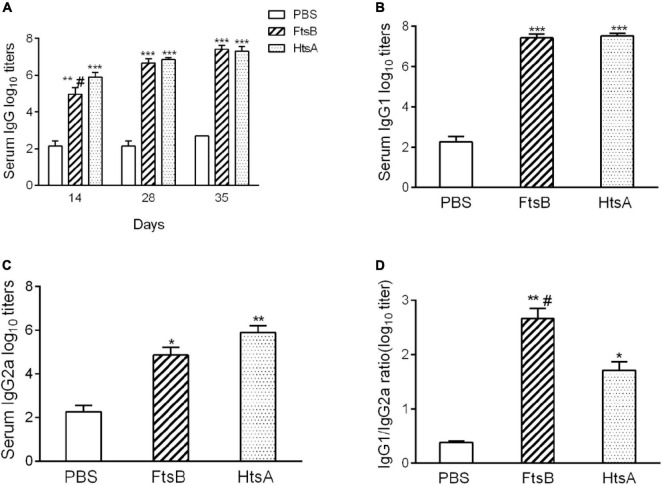
Antigen-specific antibody responses elicited by FtsB immunization. **(A)** The level of FtsB-specific IgG antibodies was determined from serum samples on days 14, 28 and 35 by ELISA. **(B)** The level of FtsB-specific IgG1 was determined from serum samples on day 35 by ELISA. **(C)** The level of FtsB-specific IgG2a was determined from serum samples on day 35 by ELISA. **(D)** The ratio of IgG1/IgG2a. *n* = 10 mice per group, *p < 0.05, **p < 0.01, ***p < 0.001 vs. PBS control group, ^#^represents *p* < 0.05 vs. HtsA group. The HtsA protein plus an aluminum adjuvant was used as a positive control, and PBS plus an aluminum adjuvant was used as a negative control.

To investigate the immunogenicity of the FtsB protein, the titers of IgG, IgG1, and IgG2a antibodies in the sera of immunized mice were detected using ELISA on post-immunization days 14, 28 and 35. Immunization with the FtsB protein elicited a high level of IgG antibody on days 14, 28, and 35, and antibody production after two boost immunizations was considerably higher than that with one immunization, suggesting that FtsB is strongly antigenic (Figure 2A). Moreover, the titers of IgG antibodies against FtsB and HtsA were similar (Figure 2A).

IgG1 and IgG2a are Th2- and Th1-associated antigen-specific IgG isotypes, which reflect the possibility of a Th2- or Th1-biased immune response, respectively. Compared to the PBS control group, both IgG1 and IgG2a antibodies were significantly induced in the FtsB-immunized mice on day 35 (Figures 2B,C). The IgG1/Ig2a ratio was greater than 2 when immunized with FtsB, indicating a Th2-biased immune response (Figure 2D).

### Immunization with FtsB reduced bacterial burden and decreased pathology in a murine bacteremia model

**FIGURE 3:**
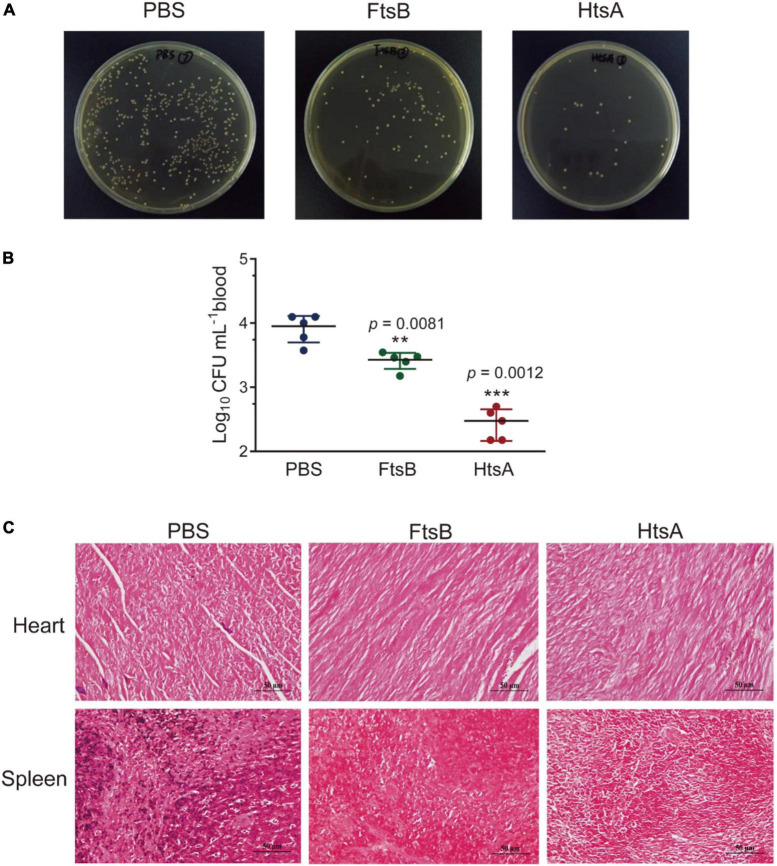
Immunization with the FtsB protein reduced the bacterial burden in a murine bacteremia model. **(A,B)** Bacterial loads in the blood of mice immunized with different antigens determined 6 h after infection with 5 × 10^6^*S. pyogenes* MGAS5005 (*n* = 5). **(C)** The bacterial loads in the heart and spleen of mice. Gram staining of the heart and spleen of mice after 48 h of challenge with *S. pyogenes* (400×). *n* = 5 mice per group, **p < 0.01, ***p < 0.001 vs. PBS control group.

We first evaluated the protective efficacy of FtsB in a murine bacteremia model. Seven days following the third immunization (day 35), mice were challenged with 5 × 10^6^ *S. pyogenes* MGAS5005. As shown in Figures 3A,B, the FtsB- and HtsA-immunized groups showed significantly lower bacterial loads in mouse blood than the PBS control group 6 h post-infection. Moreover, compared with the PBS control group, immunization with the FtsB protein decreased the bacterial burden in the heart and spleen 48 h post-infection, and similar results were also observed in HtsA-immunized mice (Figure 3C).

**FIGURE 4:**
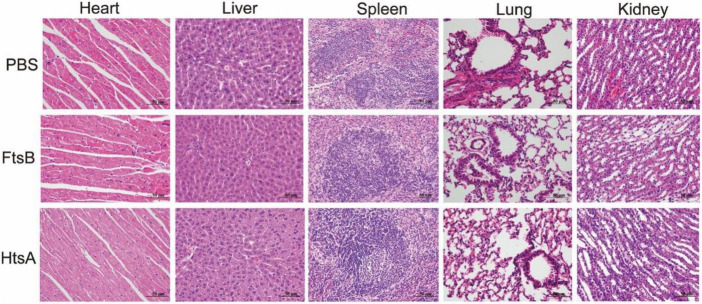
Immunization with the FtsB protein decreased the pathology of the heart, liver, spleen, lung, and kidney in a murine bacteremia model. HE images of the heart, liver, spleen, lung, and kidney in mice 48 h after challenge with 5 × 10^6^*S. pyogenes* MGAS5005 (400×).

To further observe the effect of immunization with the FtsB protein on bacteremia, histological analysis was performed. The heart, liver, spleen, lung, and kidney were collected from mice challenged with *S. pyogenes* MGAS5005 48 h after infection. The smaller cardiac-mechanical gap in the heart tissue, more neat hepatocytes arrangement in the liver, the normal and visible germinal centers in the spleen, the less alveolar damage and inflammatory cell infiltration in the lung, and the less bleeding in the kidney were observed in mice immunization with the FtsB or HtsA protein when compared with the PBS control group (Figure 4). Collectively, these data suggested that vaccinated with the FtsB protein can reduce bacterial burden and spreading in the blood, heart and spleen and reduce organ damage.

### Immunization with FtsB conferred protective immunity against *Streptococcus pyogenes* in a murine skin infection model

**FIGURE 5:**
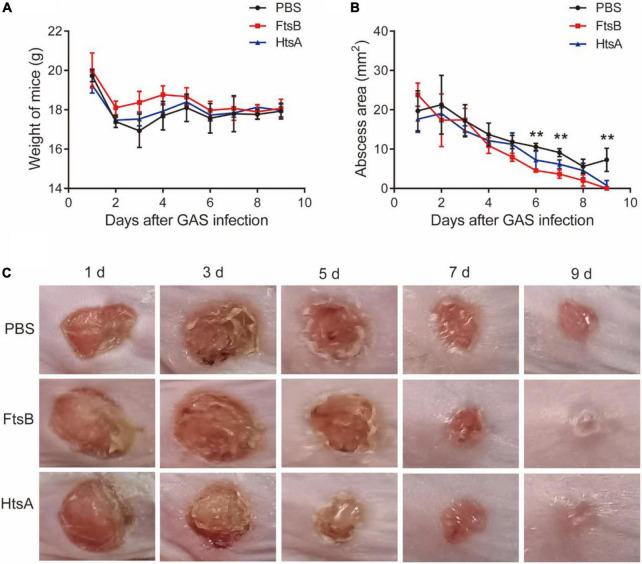
Immunization with the FtsB protein decreases the size of abscesses caused by *S. pyogenes* in a murine skin infection model. **(A)** The body weight of the mice. **(B)** The size of abscesses in mice. **(C)** Representative mouse skin lesions. *n* = 5 mice per group, **p < 0.01 represent FtsB vaccinated group vs. PBS control group.

We also used a murine skin infection model to assess the protective efficacy of FtsB against *S. pyogenes*. Seven days following the third immunization, mice were inoculated by subcutaneous injection in the shaved right flank with *S. pyogenes*. The body weight and skin abscess areas of all mice were monitored every day during a 9-day observation period. As shown in Figure 5, the body weights of mice in the vaccinated and control groups were almost unchanged; however, the *S. pyogenes* abscess sizes in mice immunized with FtsB were smaller than those in the PBS control group. Compared with the PBS control group, the skin wounds in mice immunized with FtsB or HtsA were almost healed by day 9 after infection (Figures 5B,C). In addition, there was no difference in abscess size between mice immunized with FtsB and HtsA (Figures 5B,C).

**FIGURE 6:**
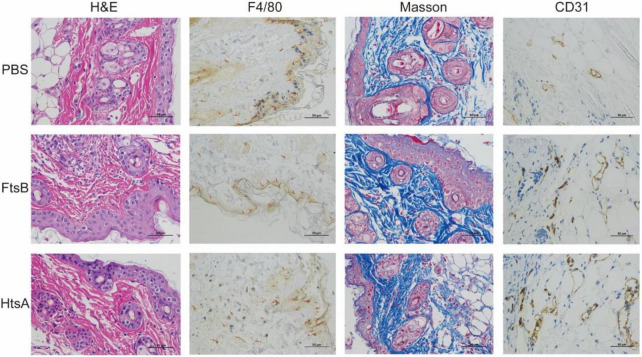
In the skin infection model, immunization with the FtsB protein reduced inflammation at the mouse skin lesions, promoted vascular endothelial regeneration and accelerated wound healing. The skin pathological conditions of the mice were monitored 48 h after challenge with *S. pyogenes*. F4/80 is a marker of macrophages, and CD31 is a marker of vascular endothelium (400×). The brown in the figures represent F4/80- or CD31-positive cells.

We further investigated the possible mechanism for FtsB-mediated protection in the skin infection model. The HE results showed that the inflammation of infected skin wounds in mice immunized with FtsB was less than that in the PBS control group (Figure 6). F4/80 and CD31 are markers of macrophages and vascular endothelium, respectively. As shown in Figure 6, compared to the PBS control group, the expression of F4/80 in skin lesions was reduced significantly in mice vaccinated with FtsB or HtsA, while the expression of CD31 was induced significantly in mice vaccinated with FtsB or HtsA. In addition, the Masson staining results indicated that collagen fibers in the infected skin wounds of the mice vaccinated with FtsB or HtsA were significantly more abundant than those in the PBS control group (Figure 6). The above results demonstrated that immunization with FtsB alleviated the severity of *S. pyogenes* skin infections and accelerated skin wound healing.

### Immunization with FtsB induced the secretion of cytokines

**FIGURE 7:**
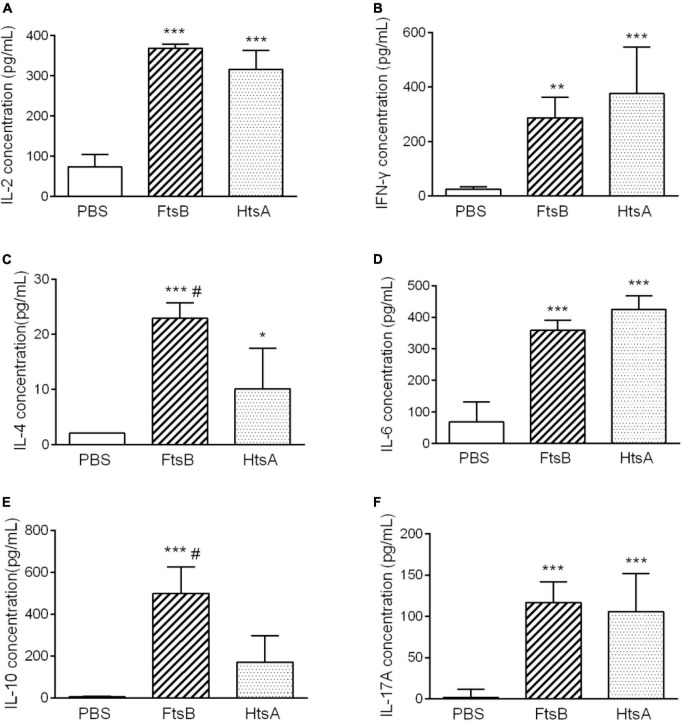
Immunization with FtsB stimulated the secretion of the cytokines IL-2, IFN-γ, IL-4, IL-6, IL-10 and IL-17A. **(A)** The secretion of the cytokine IL-2. **(B)** The secretion of the cytokine IFN-γ. **(C)** The secretion of the cytokine IL-4. **(D)** The secretion of the cytokine IL-6. **(E)** The secretion of cytokine IL-10. **(F)** The secretion of the cytokine IL-17A. *n* = 5 mice per group, *p < 0.05, **p < 0.01, ***p < 0.001 vs. PBS control group, ^#^represents *p* < 0.05 vs. HtsA group. The HtsA protein plus an aluminum adjuvant was used as a positive control, and PBS plus an aluminum adjuvant was used as a negative control.

To further investigate the possible mechanism of protective immunity initiated by FtsB, we measured the cytokine profiles of spleen cells from mice in each immunized group 7 days after the third immunization. As shown in Figure 7, elevated levels of the cytokines IL-2, IFN-γ (Th1), IL-4, IL-6, IL-10 (Th2), and the IL-17A (Th17) were observed in the FtsB- and HtsA-immunized groups compared to the PBS control group. These findings were consistent with the results from the antibody subtype assay, which suggested that immunization with FtsB or HtsA induced a strong mixed cellular immune response. Moreover, compared with the HtsA-immunized group, immunization with FtsB protein induced higher levels of IL-4 and IL-10.

### Immunization with FtsB stimulated dendritic cell maturation

**FIGURE 8:**
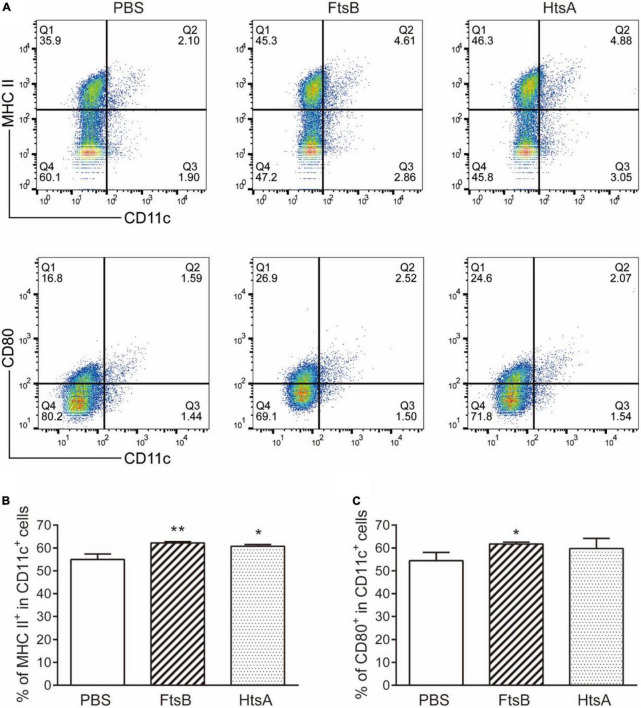
Immunization with FtsB stimulated the maturation of DCs. **(A)** Representative flow cytometry graph. **(B)** The percentage of the costimulatory molecule MHCII expressed on CD11c + DC cells. **(C)** The percentage of the costimulatory molecule CD80 expressed on CD11c + DCs. *n* = 5 mice per group, *p < 0.05, **p < 0.01 vs. PBS control group.

To investigate the effect of immunization with FtsB protein on the maturation of DCs, we measured the expression of the MHC II and CD80 molecules on CD11c^+^ DCs from murine spleen cells in each immunized group by using flow cytometry. Compared with the PBS control group, the percentage of MHC II and CD80 molecule expression by CD11c^+^ DCs in mice vaccinated with FtsB or HtsA was significantly increased (Figure 8). These results indicated that immunization with FtsB can improve the maturation of DCs in mice, which is beneficial for inducing more effective antigen presentation and immune responses.

## Discussion

*S. pyogenes* is one of the major pathogens responsible for human disease, and iron plays an important role in its growth and infection. Iron uptake systems are the major virulence determinants for many pathogens, and their lipoproteins are exposed on the extracellular wall and thus have been proven to be promising vaccine candidates in many pathogens (Cassat and Skaar, 2013; Nguyen and Gotz, 2016; Kramer et al., 2020). In *S. pyogenes*, there are three iron uptake systems involved in iron uptake, HtsABC, FtsABCD and MtsABC, with the lipoproteins HtsA, FtsB and MtsA, respectively (Janulczyk et al., 2003; Lei et al., 2003; Hanks et al., 2005). It has been reported that the HtsA protein can be used as a vaccine candidate to protect against *S. pyogenes* infection (Song et al., 2020). Immunization with the HtsA protein can reduce the invasion of *S. pyogenes*, improve the survival rate, and reduce the size of skin lesions in mice (Song et al., 2020). However, whether the FtsB protein can serve as an *S. pyogenes* vaccine candidate has not yet been reported.

In the present study, we obtained the FtsB protein with high purity and thoroughly evaluated the immunogenicity it induced in BALB/c mice, as well as its protection against *S. pyogenes* infection. Immunization with the FtsB protein induced the production of high titers of antigen-specific IgG, IgG1 and IgG2a in mice. Moreover, the FtsB protein was able to induce a protective immune response against *S. pyogenes* infection in a murine bacteremia model and a skin infection model. In the murine bacteremia model, a low *S. pyogenes* bacterial load was recovered from the blood, heart, and spleen, and less organ damage, including less damage to the heart, liver, spleen, lung, and kidney, was observed in FtsB-vaccinated mice than in the PBS control group. In the murine skin infection model, there were smaller skin abscess areas and fewer inflammatory skin infection wounds in mice vaccinated with FtsB than in the PBS control group. These results indicate the protective efficacy of the FtsB vaccine.

In addition, we dissected the possible mechanisms of protection induced by FtsB from two perspectives: cytokine production and DC activation. Spleen cells from mice immunized with antigens can be stimulated *in vitro* to secrete specific cytokines that play important roles in antigen-specific immune responses. Cytokines play a key role in protecting the host from bacterial infection by modulating the innate immune response (Soderholm et al., 2018). DCs play a key role in activating innate immune cells and initiating adaptive immune responses, and they are also intermediate regulators responsible for sensing pathogens and activating effectors to eliminate them (Durai and Murphy, 2016). In this study, the protective effects of FtsB were accompanied by the secretion of IL-2, IFN-γ, IL-4, IL-6, IL-10, and IL-17A by stimulated splenocytes, as well as the maturation of DCs.

It has been reported that the HtsA protein can be used as a vaccine candidate against *S. pyogenes* infection (Song et al., 2020). Here, the IgG, IgG1 and IgG2a titers, the protective effects against *S. pyogenes* infection in two murine models, the levels of IL-2, IFN-γ, IL-6, and IL-17A production, and the percentage CD11c^+^ DCs expressing the MHC II and CD80 molecules were similar in mice immunized with FtsB protein compared to the HtsA positive control. However, FtsB induced an increased IgG1/Ig2a ratio, and the levels of the Th2-type cytokines IL-4 and IL-10 were higher than those of HtsA, which indicated that FtsB induced a Th2-biased immune response and can serve as an *S. pyogenes* vaccine candidate. On the other hand, compared to the M protein-based vaccine candidates, e.g., J8-DT (Pandey et al., 2015), the protective effect of the FtsB vaccination is weak, suggesting that the improvement and optimization of FtsB vaccine needs to be done in the future.

## Conclusion

In conclusion, immunization with the FtsB protein could elicit immune responses, including the induction of high antigen-specific IgG, IgG1, and IgG2a titers, the secretion of the cytokines IL-2, IFN-γ, IL-4, IL-6, IL-10, and IL-17A, and the maturation of DCs, leading to protective immunity in a murine model. These results provide evidence for the FtsB protein as a vaccine candidate against *S. pyogenes* infection.

## Data availability statement

The raw data supporting the conclusions of this article will be made available by the authors, without undue reservation.

## Ethics statement

This animal study was reviewed and approved by the Committee on the Use of Live Animals in Teaching and Research of Zunyi Medical University.

## Author contributions

L-YH and Y-BY designed the research, performed the experimental work, and wrote the manuscript. YL and Y-JL analyzed the data. X-YY and SL provided the initial idea, designed the research, and revised the manuscript. All authors contributed to manuscript revision and read and approved the submitted version.
