# Supplementary material for: Immunization with the lipoprotein FtsB stimulates protective immunity against Streptococcus pyogenes infection in mice
Source: Front Microbiol. 2022 Aug 9;13:969490. doi: 10.3389/fmicb.2022.969490 (PMC9396372; doi:10.3389/fmicb.2022.969490)
Supplement: Supplementary file 1 [file Table_1.docx]

**Table S1 Amino acid identities and similarities of FtsB protein of various bacteria to *Streptococcus pyogenes* MGAS5005 FtsB protein.**

| **Bacteria** | **Accession numbers** | **Identities with FtsB** | **Positives with FtsB** |
| --- | --- | --- | --- |
| *Streptococcus pyogenes* GA40468 | ESA47425.1 | 99% | 99% |
| *Streptococcus dysgalactiae subsp. equisimilis* SK1249 | EGL49540.1 | 84% | 92% |
| *Streptococcus equi subsp. zooepidemicus* Sz12is | KIS04800.1 | 82% | 90% |
| *Streptococcus parauberis* KCTC 11537 | AEF25782.1 | 74% | 86% |
| *Streptococcus sp*. DD10 | WP_156468545.1 | 65% | 84% |
| *Streptococcus sanguinis* SK150 | EGD36994.1 | 55% | 76% |
| *Streptococcus suis* R61 | EHC02854.1 | 43% | 69% |
| *Planomicrobium sp*. CPCC 101079 | WP_146494059.1 | 42% | 65% |
| *Paenibacillus sp*. P22 | WP_048748158.1 | 43% | 64% |
| *Bacillus sp.* PDNC022 | WP_205184277.1 | 41% | 62% |
| *Psychrobacillus sp*. INOP01 | WP_211892925.1 | 40% | 62% |
| *Sporosarcina sp*. BI001-red | WP_116017071.1 | 40% | 62% |
| *Bacillus cereus group sp*. BfR-BA-01379 | WP_242253223.1 | 39% | 62% |
| *Clostridium sp*. HMP27 | KGK84185.1 | 37% | 62% |
| *Listeria sp*. PSOL-1 | WP_163652061.1 | 38% | 61% |
| *Viridibacillus sp*. JNUCC-6 | WP_193737955.1 | 40% | 60% |
| *Staphylococcus sp*. K22-5M | WP_208181734.1 | 39% | 60% |
| *Fictibacillus sp*. FJAT-27399 | WP_234293365.1 | 39% | 60% |
| *Niallia sp*. NCCP-28 | WP_248562394.1 | 38% | 60% |
| *Sporolactobacillus sp*. THM7-7 | WP_130000326.1 | 40% | 59% |
| *Planococcus sp*. NCCP-2050 | WP_251476806.1 | 39% | 59% |
| *Fictibacillus sp*. KIGAM418 | WP_248251236.1 | 38% | 59% |
| *Anoxybacillus sp*. MB8 | WP_163150686.1 | 38% | 59% |
| *Escherichia coli* K-12 | P0AEL6.1 | 23% | 42% |
